# Editorial: Multidisciplinary aspects and performance in racket sports

**DOI:** 10.3389/fpsyg.2024.1427362

**Published:** 2024-05-20

**Authors:** Rafael Martínez-Gallego, Bernardino Sánchez-Alcaraz, Goran Vučković, Jesus Ramón-Llin

**Affiliations:** ^1^Department of Physical Activity and Sport, University of Valencia, Valencia, Spain; ^2^Department of Physical Activity and Sport, University of Murcia, Murcia, Spain; ^3^Faculty of Sport, University of Ljubljana, Ljubljana, Slovenia; ^4^Department of Didactics of Physical Education, Artistic and Music, University of Valencia, Valencia, Spain

**Keywords:** table tennis, padel player, badminton, tennis, physical fitness, psychological dynamics, physiological demands, technical skills

The convergence of disciplines such as exercise physiology, sport psychology, biomechanics, and nutrition has proven to be fundamental in enhancing sport performance (Hanton, 2006; Jeukendrup and Gleeson, 2019). By integrating knowledge and techniques from different fields, a deeper understanding of the factors that influence sporting success is achieved, allowing for the design of comprehensive strategies that maximize performance and prevent injury (Kraemer and Fleck, 2007). This interdisciplinary approach not only improves physical variables such as strength and endurance (Baechle and Earle, 2008), but also affects psychological aspects such as motivation and stress management, thus promoting sporting excellence in a holistic and sustainable manner (Williams, 1993).

Thus, in this Research Topic of Frontiers in Psychology, titled “*Multidisciplinary Aspects and Performance in Racket Sports*” we explore a comprehensive range of studies that elucidate the complex balance of physical fitness, psychological dynamics, physiological demands, and technical skills across various racket sports. This editorial aims to connect together the findings from these diverse studies, highlighting their unified implications for athletes, coaches, and sports science professionals.

Physiological demands across different racket sports are systematically reviewed by Cádiz Gallardo et al., emphasizing sport-specific training needs and health benefits. This study serves as a crucial reference for tailoring athlete training regimens according to the unique demands of each sport. Following the topic of physical and physiological aspects, an innovative study by Pradas de la Fuente et al., provides a detailed assessment of physical fitness in young high-level table tennis players. This research not only outlines differences based on sex, age, and playing style but also sets a precedent for how such data can guide targeted training programs, potentially developing performance and career longevity in table tennis. Related with these aspects, the innovative work by Zhou et al. in the field of training methodology examines the effects of combined balance and plyometric training on knee function and proprioception in elite badminton players. This study not only expands the understanding of effective training techniques but also highlights the importance of comprehensive training regimens that address multiple aspects of athlete development.

Parallel to physical fitness, psychological readiness plays a pivotal role in sports. Conde-Ripoll et al. investigate precompetitive anxiety and self-confidence among high-level men's padel players. Their findings underscore the importance of psychological management in achieving peak performance, particularly how anxiety levels fluctuate with competition stages and outcomes, while self-confidence remains comparatively stable. In a related manner, Castillo-Rodriguez et al. expand on the psychological aspects by examining how playing category, BMI, and experience influence precompetitive anxiety and self-confidence in padel players. Their study reveals that higher categories are associated with higher self-confidence and lower somatic anxiety, indicating that psychological traits could be as critical as physical skills in determining sports performance.

From a technical perspective, Pradas et al. provide interesting insights into the sex differences in serve strategies and returns among elite table tennis players. Their work suggests that these differences may reflect broader physiological and psychological variations, which could inform more personalized coaching strategies. Also, on the technical side of youth training, Touzard et al. investigate the effects of racket scaling on serve biomechanics in young tennis players. Their findings support for a cautious approach to equipment scaling, which is vital for optimizing development and minimizing injury risks. The development of young athletes was also explored by He et al., investigating how family background influences self-efficacy in adolescent table tennis players, with technical learning engagement playing a mediating role. This study suggests that early interventions aimed at enhancing engagement can be particularly beneficial.

Turning the focus to the officiating side of sports, Li and Li's study on tennis officials in China addresses job satisfaction and turnover intentions, pointing to the critical need for supportive structures that promote a sense of community and motivation. This highlights an often-overlooked aspect of sports ecosystems, where the wellbeing of officials can indirectly influence the quality of the sport itself.

Together, these studies offer a rich understanding of insights that reinforce the multidimensional nature of racket sports. They collectively advance our understanding of how physical and psychological factors interact to structure athlete performance in these dynamic sports. As we continue to uncover these complex interactions, our strategies for training, competition, and management in racket sports will undoubtedly evolve, promising enriched outcomes for athletes at all levels.

## Author contributions

RM-G: Writing – original draft, Writing – review and editing. BS-A: Writing – original draft, Writing – review and editing. GV: Writing – original draft, Writing – review and editing. JR-L: Writing – original draft, Writing – review and editing.
